# Pleistocene sea level fluctuation and host plant habitat requirement influenced the historical phylogeography of the invasive species *Amphiareus obscuriceps* (Hemiptera: Anthocoridae) in its native range

**DOI:** 10.1186/s12862-016-0748-3

**Published:** 2016-08-31

**Authors:** Danli Zhang, Zhen Ye, Kazutaka Yamada, Yahui Zhen, Chenguang Zheng, Wenjun Bu

**Affiliations:** 1 Institute of Entomology, College of Life Sciences, Nankai University, 94 Weijin Road, Tianjin, 300071 China; 2 Tokushima Prefectural Museum, Bunka-no-Mori Park, Hachiman-chô, Tokushima 770-8070 Japan

**Keywords:** Dispersal ability, East China Sea, Cological niche modeling, Genetic structure, Habitat requirement, Last Glacial Maximum, Land bridge

## Abstract

**Background:**

On account of repeated exposure and submergence of the East China Sea (ECS) land bridge, sea level fluctuation played an important role in shaping the population structure of many temperate species across the ECS during the glacial period. The flower bug *Amphiareus obscuriceps* (Poppius, 1909) (Hemiptera: Anthocoridae) is an invasive species native to the Sino-Japanese Region (SJR) of East Asia. We tested the hypothesis of the ECS land bridge acting as a dispersal corridor or filter for *A. obscuriceps* during the glacial period. Specifically, we tested whether and the extent to which dispersal ability and host plant habitat requirement influenced the genetic structure of *A. obscuriceps* during the exposure of the ECS land bridge.

**Results:**

Phylogenetic and network analyses indicated that *A. obscuriceps* is composed of two major lineages, i.e., China and Japan. Divergence time on both sides of the ECS was estimated to be approximately 1.07 (0.79–1.32) Ma, which was about the same period that the sea level increased. No significant Isolation by Distance (IBD) relationship was found between *Фst* and Euclidean distances in the Mantel tests, which is consistent with the hypothesis that this species has a good dispersal ability. Our Last Glacial Maximum (LGM) niche modeling of plants that constitute preferred habitats for *A. obscuriceps* exhibited a similar habitat gap on the exposed ECS continental shelf between China and Japan, but showed a continuous distribution across the Taiwan Strait.

**Conclusion:**

Our results suggest that ecological properties (habitat requirement and dispersal ability), together with sea level fluctuation during the Pleistocene across the ECS, have shaped the genetic structure and demographic history of *A. obscuriceps* in its native area. The host plant habitat requirement could also be a key to the colonization of the *A. obscuriceps* species during the exposure of the ECS land bridge. Our findings will shed light on the potential role of habitat requirement in the process of biological invasion in future studies.

**Electronic supplementary material:**

The online version of this article (doi:10.1186/s12862-016-0748-3) contains supplementary material, which is available to authorized users.

## Background

The eastern areas of Sino-Japanese region stretch along more than 1500 km in an east–west direction, ranging from East China to Japan and the Korean Peninsula to Taiwan and the Ryukyu Islands [1]. Historically, this region has been heavily influenced by glacial–interglacial cycles during the Pleistocene, showing variations of coastal habitat availability and geomorphological patterns along the rim of the northwest Pacific [2]. During the LGM (approximately 22,000 years ago) of the last glacial period, wide extensions of continental shelf across the ECS were assumed to have been exposed, forming a large land bridge connecting the isolated continents of Mainland China, Japan, Taiwan, and probably the Korean Peninsula, because the sea levels were 85–140 m below the present levels [3]. Fossil pollen analyses and paleobiome reconstructions also indicate that several temperate plant species expanded across large areas of the continental shelf that emerged in the ECS as a consequence of sea level decline during the cold periods [4]. Phylogeographic structure and Pleistocene history of temperate species inhabiting the SJR were closely linked to the incidents of historical climatic changes associated with sea level fluctuations and land bridge configuration. The ECS land bridge, along with the sea level decline during the LGM, might have connected species or population distribution, either serving as a dispersal corridor, allowing continuous migration of many temperate species from the mainland China into Japan (or vice versa), or generating secondary contact or gene flow among the formerly isolated populations. This “dispersal corridor” hypothesis is supported by the extant distribution of several species, including the deciduous orient oak *Quercus variabilis* Blume, 1850 [5], the marine gastropod *Thais clavigera* (Küster, 1860) [6], and the semiaquatic insect *Microvelia douglasi douglasi* Scott, 1874 [7]. In these examples, a nearly homogenized genetic pattern was observed in the chloroplast/mitochondria/nuclear DNA sequences on both sides of the ECS land bridge (i.e., Mainland China and Japan). These observations are consistent with the large expanses of suitable spaces across the ECS land bridge reconstructed by paleoclimate niche modeling [5, 7].

In contrast, many studies provided evidence that the ECS land bridge also served as a dispersal filter that obstructed or even inhibited certain species or populations from migrating through the ECS land bridge during the LGM, as seen for two deciduous trees *Platycrater arguta* Siebold & Zucc., 1835 [8] and *Acer mono* Maxim, 1856 [9]. This phenomenon suggests a high level of lineage differentiation on both sides of the ECS land bridge. A general interpretation of this genetic partition is that environment of the exposed ECS basin area was unsuitable for the survival of these species based on the evidence of LGM paleoclimate niche modeling reconstruction, which indicated that the ECS land bridge probably imposed a formidable barrier to dispersal during the glacial period [8]. However, this divergence interpretation emphasizes the external environment (i.e., glacial condition) but ignores the ecological properties of the species, including their dispersal abilities, habitat requirements, life histories and other factors. In particular, dispersal ability and habitat requirement are expected to have a strong influence on shaping the current population genetic structure [10]. Recent studies showed that, even if the suitable habitat was fragmented or discontinuous in some species distribution ranges, such geographical limitations could be overcome by species with moderate/strong dispersal abilities [11]. Many small animal species were also assumed to depend on their microhabitats, which could elude predators and supply adequate food and breeding places. This habitat requirement might restrict the species dispersal, shape a specific genetic structure, and even potentially derive new taxon from different lineages. For instance, studies on the reef-associated surgeon-fish, genus *Acanthurus* and the rock-associated lizard, genus *Dalmatolacerta*, indicate that habitat requirement is the main factor that shaped different phylogeographic patterns in these habitat-associated species [12, 13].

Few studies are focused on temperate invertebrate species of comprehensive samples, and the influence of dispersal ability and habitat requirement on the population genetic structure across the ECS are poorly understood. In this study, we focus on the flower bug *Amphiareus obscuriceps* (Poppius, 1909) (Hemiptera: Anthocoridae), a native species across the ECS land bridge [14, 15]. This species can be found in various plant microhabitats where they prey on small anthropods, principally inhabiting in the dead leaf clusters of deciduous trees and shrub [14]. In its native range, most *A. obscuriceps* were usually collected from the deciduous trees, *Castanea mollissima* Blume, 1851, *Salix babylonica* L., 1753, *Salix matsudana* Koidz, 1915, and the shrub, *Sorbaria sorbifolia* (L.) A. Braun, 1860 [14], all of which are native to East Asia [16]. Recently, *A. obscuriceps* has become an invasive species that is rapidly spreading in North America and Europe [15, 17, 18]. Considering its host plant habitat requirement and potentially moderate/strong dispersal ability like other invasive species, *A. obscuriceps* provides an ideal model for examining the phylogeographic structure and Pleistocene history associated with sea level fluctuation and ecological property across the ECS during the glacial period. We used a comprehensive sampling of *A. obscuriceps* in its native area, and applied ecological niche modeling (ENM) and multilocus genetic markers to infer a more robust phylogeographic conclusion of this species across the ECS. Specifically, this study aimed to (1) describe the phylogeographic structure; (2) infer the divergence time and historical demography; (3) test the hypothesis of ECS land bridge as a dispersal corridor or filter for *A. obscuriceps* in its native area during the last glacial period; and (4) evaluate the role of habitat requirement and dispersal ability in driving the current genetic structure during the exposure of the ECS land bridge in LGM period.

## Methods

### Sampling and laboratory procedures

**Fig. 1:**
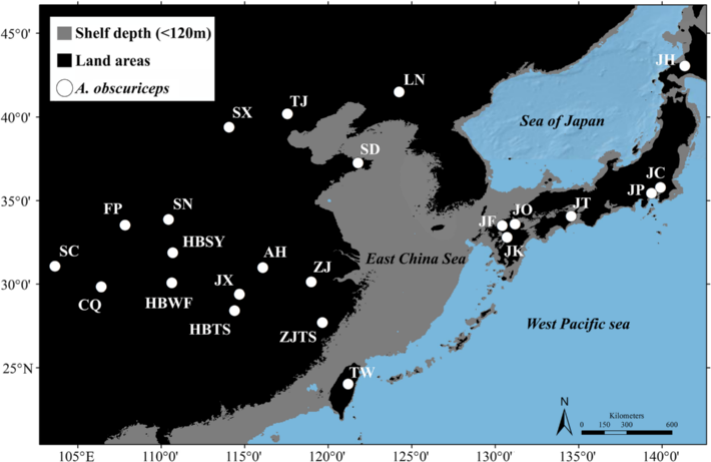
Map showing sample locations of *A. obscuriceps*. Samples are marked by abbreviations that correspond to Table 1. Shaded sea areas are continental shelves that would have been exposed to the air during periods of lower sea-levels (LGM)

We sampled 23 populations, comprising a total of 188 individuals in its native areas covering Mainland China, Taiwan Island and Japan (Fig. 1). *Amphiareus ruficollaris* Yamada & Hirowatari, 2003 was used as the outgroup for our phylogenetic analysis. All samples were preserved in 95 % ethanol and stored in a freezer at −20 °C in the College of Life Sciences at Nankai University (Tianjin, China). Genomic DNA was extracted from the entire body, excluding the abdomen and genitalia using a General AllGen Kit (Qiagen, Germany). All individuals were analyzed by sequencing for the mitochondrial markers, cytochrome c oxidase subunit I (COI), cytochrome c oxidase subunit II (COII), and cytochrome b (CytB) and the nuclear marker internal transcribed spacer 1 (ITS1). PCR primers designed in the present study are listed in Table S1 (Additional file 1: Table S1). The PCR program included an initial denaturation at 94 °C for 2 min, followed by 31–33 cycles of 30 s at 92 °C, 30 s at 50–52 °C, and 1 min at 72 °C, ending with a final extension at 72 °C for 8 min. PCR products were sent to BGI (Beijing Genomics Institute) where sequencing was conducted in both directions using a HiSeq 2000 sequencing system. Sequences were aligned with ClustalW [19] multiple alignment under default settings and visually proofread in Bioedit v7.0 software [20].

### Phylogenetic analysis

Gene tree reconstructions of concatenated mitochondrial haplotypes were performed using Bayesian inference (BI) and Maximum likelihood (ML) methods, and implemented in MrBayes 3.2.5 [21] and raxmlGUI 1.5 [22] respectively. The program PartitionFinder 1.1.1 [23] was used to infer the best partitioning strategy and models of substitution for each gene in Bayesian and ML analyses. We used the Akaike information criterion (AIC) and the “greedy” algorithm with branch lengths estimated as “unlinked” to search for the best-fit scheme. For the Bayesian analysis, MrBayes 3.2.5 [21] was employed to reconstruct the phylogenetic trees under the HKY substitution model with a gamma distribution (+ G) and a proportion of invariable sites (+ I) to account for among-site rate variation. Two simultaneous runs of 10,000,000 generations were conducted for concatenated matrix. Trees inferred prior to stationarity (at 25 % of the run) were discarded as burn-in, and the remaining trees were used to construct a 50 % majority-rule consensus tree. ML analysis was conducted with raxmlGUI 1.5 [22] under the substitution model GTRGAMMAI and the node support values were assessed by bootstrap resampling calculated using 1000 replicates.

### Genetic diversity and phylogeographic analysis

Genetic diversities of mitochondrial and nuclear data were estimated for all populations and for each population using the number of polymorphic sites (*S*), number of haplotypes (*Nhap*), haplotype diversity (*Hd*), and nucleotide diversity (*π*), which were all calculated in DNASP 4.0 [24]. The algorithm of haplotype generation considered the sites with gaps/missing data. Network 4.6.1.3 (Fluxus Technology, Suffolk, UK) was used to create intraspecific median-joining networks to visualize the evolutionary relationships between haplotypes [25]. We also assessed the hierarchical partitioning of genetic structure between specimens from China and those from Japan, based on K2P distance by analyzing molecular variance (AMOVA) in Arlequin 3.5 [26]. Only the populations with more than 5 individuals were analyzed. The Mantel test was conducted in Isolation by Distance Web Service (IBDWS) using 1000 randomizations to test the isolation-by-distance model [27]. Finally, the potential geographical zones associated with genetic discontinuities across the entire sampling regions were further investigated by using Monmonier’s algorithm implemented in Barrier 2.2 [28].

### Coalescent inferences of demographic history

Demographic history was initially examined using two different approaches. First, mismatch distribution was used to identify recent demographic expansion and population equilibrium processes [29]. Mismatch distribution defined groups of populations by haplotype networks, and the statistical charts were calculated based on a population growth–decline model in DNASP 4.0 [24]. Then, three neutrality tests, Tajima’s *D*, Fu and Li’s D*, and Fu’s FS, which were implemented in DNASP 4.0 [24] or ARLEQUIN 3.5 [26], were performed. Extended Bayesian skyline plots (EBSP) were implemented in BEAST 1.8.2 [30] to estimate the shape of population growth through time with coupled multilocus data, which reduced estimation errors associated with single gene and increased the power of detecting demographic dynamics [31]. Analyses were conducted across the multilocus datasets (COI, CytB, and ITS1) by using a relaxed uncorrelated lognormal molecular clock. Given that the mutation rates for this species are unknown, we used the following confidence intervals from Reduviidae (Hemiptera: Cimicomorpha): COI, 0.6–1.0 %/Ma [32, 33]; CytB, 1.1–1.8 %/Ma [34]; and ITS1, 0.4–1.0 %/Ma [35]. The data set was partitioned by genes and Modeltest was used to determine the best-fit model under the AIC for each gene [36]. We ran the chains for 100,000,000 generations, and gene trees were saved every 1000 generations. EBSP coalescent model using linear type and the ploidy type had been properly setting. Convergence of the Markov Chain Monte Carlo (MCMC) chains was inspected using Tracer 1.6 [37] by visually checking that the effective sample size (ESS) for all relevant parameters were well above 200.

### Estimation of divergence time and migration rate

We estimated the divergence time (*t*) and migration rate (*m*) using the isolation-with-migration (IM) model implemented in IMa2 [38]. In contrast to BEAST, the Bayesian approach in IMa2 generated a more realistic model because it considered possible gene flows among populations after they split from an ancestral species [39]. We used mitochondrial sequences for the IM analysis. The three component groups (i.e., Mainland China, Japan, and Taiwan) were divided according to the results of their haplotype networks. We initially conducted multiple short runs with an increasing number of steps, wide priors, and heating schemes to ensure that the complete posterior distribution could be obtained. The HKY model with 0.25 as the inheritance scalar was used, and the upper prior bounds were set to 20 for the population size (*q*), 10 for the divergence time (*t*), and 1 for the migration rate (*m*). Parameters were estimated based on mutation rates for COI (0.6 × 10^−8^–1.0× 10^−8^) and CytB (1.1 × 10^−8^–1.8× 10^−8^) per site per generation [32–34], and average generation time was set to 0.5 years according to previous studies of other Anthocoridae species [40]. Final runs consisted of 1 × 100,000,000 steps with a burn-in of 1 × 10,000,000 steps, and the lowest ESS among the parameters was greater than 50. In addition, we also used BEAST 1.8.2 [30] to estimate divergence time. The data set was partitioned by genes and was set specific clock per gene (COI and CytB). We used a coalescent exponential growth model with the chains run for 100,000,000 generations, which was checked to make sure the ESS was more than 200 and the first 10 % discarded as burn-in.

### Paleoclimate niche modeling reconstruction

A total of 52 occurrence records of *A. obscuriceps* in its native area were used for niche modeling, including 23 records from the alcohol-preserved specimens and 29 records from pinned specimens deposited in Nankai University or the published literature. Model geographic background was a squared area (Y max = 45, Y min = 17, X max = 145, X min = 100) delimited by a bounding box containing all known native range occurrences of *A. obscuriceps* typically used in former studies [41]. Meanwhile, we also used host plants *C. mollissima*, *Sa. babylonica*, *Sa. matsudana* and *So. sorbifolia* [14] for niche modeling to examine the degree to which the distribution of the related host plant species of *A. obscuriceps* have changed since the Pleistocene glaciation. The host plant coordinates in its native area for niche model building were downloaded from the Global Biodiversity Information Facility online database (GBIF, http://www.gbif.org/), which determined 335 localities for *C. mollissima*, 93 for *Sa. babylonica*, 59 for *Sa. matsudana*, and 44 for *So. sorbifolia*. We chose bioclimatic variables to represent annual trends and extreme or limiting conditions, because many taxa are limited by environmental extremes [41]. Seven bioclimatic variables, which were most likely to restrict the distribution of *A. obscuriceps* and related host plants, were selected from the WorldClim database [42] (http://www.worldclim.org/), including mean temperature (BIO1), mean diurnal temperature range (BIO2), maximum temperature of the warmest month (BIO5), minimum temperature of the coldest month (BIO6), annual mean precipitation (BIO12), precipitation of the wettest month (BIO13), and precipitation of the driest month (BIO14). We conducted an autocorrelation test of the 7 bioclimate variables that showed relatively low Spearman’s coefficients (*r* < 0.80) (Additional file 2: Table S2). All the variables were derived from the WorldClim data centre at a resolution of 2.5-arc. We used the maximum entropy implemented in Maxent 3.3.3 k [43] to develop the current distribution model at 2.5 arc-min resolution. The analysis was run using the default program conditions, with 75 % of the recorded species for training and 25 % for testing the model. The model was then projected onto the set of climatic variables simulated by the Community Climate System Model 3 [44] to infer the extent of suitable areas during the LGM. The areas under the curve (AUC) of the “receiver operating characteristic (ROC)” plot were used for model evaluation [45]. The same seven environmental variables were used to assess global areas of potential invasion. We used 52 occurrences of the native localities for model construction and applied their prediction onto the invasive continents. We compared areas of potential invasion with the actual records in Europe and North America for model evaluation. Discoveries of *A. obscuriceps* in different countries were summarized, and 135 actual invasion records in the US and Europe were obtained from published studies (Additional file 3: Table S3). In addition, we also collected all the coordinates of the four host plants from the GBIF outside the native range, and positioned the actual records to the map of the predicted *A. obscuriceps* invasive areas.

## Results

### Genetic diversity and phylogenetic analysis

For mitochondrial DNA, 1584 bp of protein-coding regions were obtained from 188 individuals, including segments of the COI (633 bp), COII (540 bp) and CytB (411 bp) genes. A total of 182 unique haplotypes were derived from tandem sequence (COI + COII + CytB) among all individuals. The 277 polymorphic sites included 107 singleton variables and 170 parsimony informative sites. The nucleotide and haplotype diversities ranged from 0.00168 to 0.01199 (mean 0.01926) and from 0.667 to 1.000 (mean 0.9995) respectively (Table 1). Both BI and ML phylogenetic analyses of *A. obscuriceps* included two major lineages (i.e., China and Japan) (Additional file 4: Figure S1 and Additional file 5: Figure S2). Individuals from Taiwan were structurally nested within the Chinese lineage (Additional file 4: Figure S1 and Additional file 5: Figure S2).

**Table 1:** Nucleotide polymorphisms in each geographic population

COI + COII + CytB	Lat.	Long.	Sample size	*S*	*Nhap*	*Hd*	*π*
Mainland China							
AH	30°58’57”	116°4’49”	9	46	9	1.000	0.00838
CQ	29°50’16”	106°23’48”	10	47	10	1.000	0.00770
FP	33°31’51”	107°49’45”	10	40	10	1.000	0.00650
HBSY	31°52’49”	110°41’13”	2	19	2	1.000	0.01199
HBTS	29°23’57”	114°40’51”	1	–	1	–	–
HBWF	30°4’40”	110°37’33”	1	–	1	–	–
JX	28°25’9”	114°23’3”	9	41	9	1.000	0.00733
LN	41°29’51”	124°14’56”	12	45	12	1.000	0.00697
SC	31°4’5”	103°37’49”	10	39	10	1.000	0.00647
SD	37°14’34”	121°46’41”	9	22	9	1.000	0.00454
SN	33°52’29”	110°25’54”	10	43	10	1.000	0.00735
SX	39°23’2”	114°3’40”	12	46	12	1.000	0.00962
TJ	40°11’16”	117°33’22”	4	10	4	1.000	0.00358
ZJ	30°7’54”	118°59’4”	14	32	13	0.989	0.00572
ZJTS	27°42’29”	119°39’4”	8	32	8	1.000	0.00780
Japan							
JC	35°47’15”	139°54’11”	9	49	9	1.000	0.00775
JF	33°29’58”	130°25’19”	10	35	10	1.000	0.00540
JH	43°3’44”	141°21’16”	6	29	6	1.000	0.00648
JK	32°48’11”	130°42’28”	9	37	9	1.000	0.00614
JO	33°35’54”	131°11’18”	10	32	10	1.000	0.00452
JP	35°26’35”	139°21’45”	3	4	2	0.667	0.00168
JT	34°4’13”	134°33’17”	10	36	10	1.000	0.00574
Taiwan							
TW	24°1’22”	121°11’14”	10	51	10	1.000	0.01072

*S* number of segregating sites, *NHap* number of haplotypes, *Hd* haplotype diversity, *π* nucleotide diversity

### Phylogeographic analysis

**Fig. 2:**
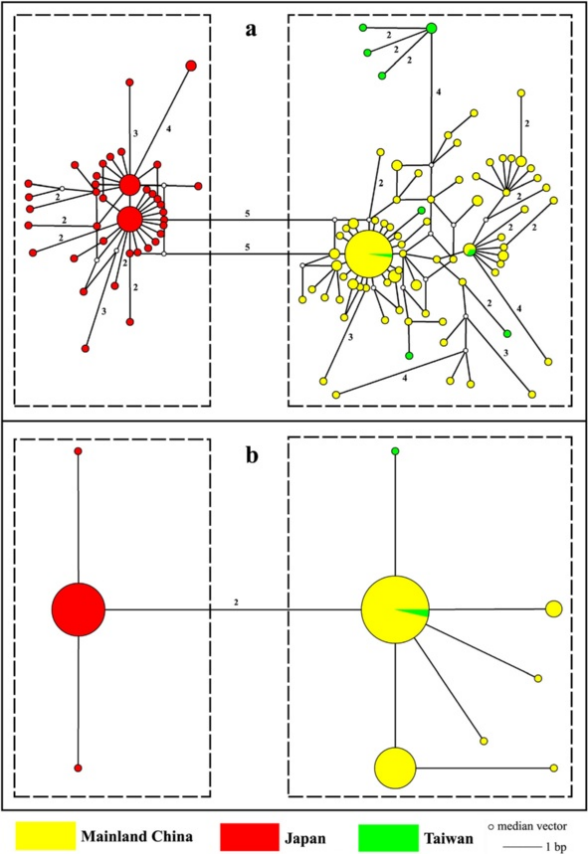
Median joining haplotype network constructed using Network. Haplotype circle size denotes the number of sampled individuals. Colors correspond to different regions. Numbers of base pair changes (no number = 1 bp) are given. **a** Mitochondrial data. **b** Nuclear data

**Fig. 3:**
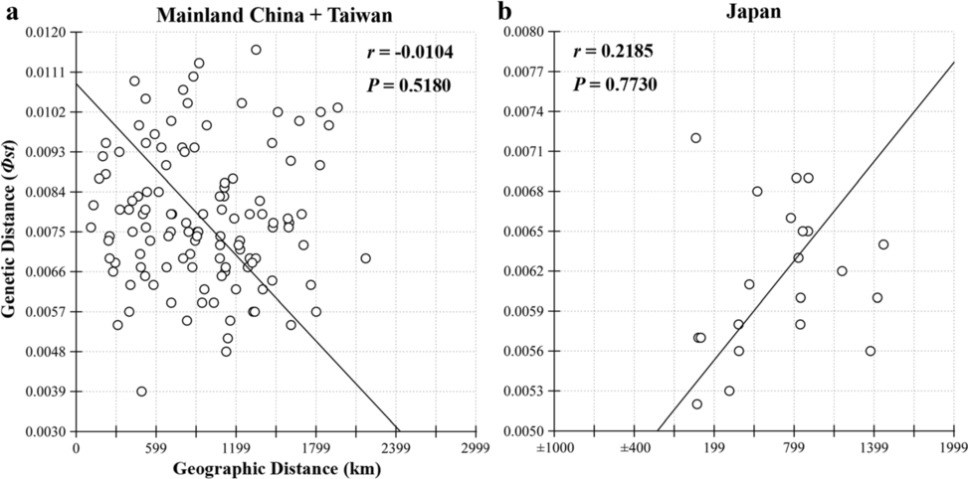
Scatter plot showing the relationship between genetic distances (Фst) and geographical distances (km). **a** Lineages of Mainland China and Taiwan. **b** Lineage of Japan

When the 182 haplotypes were used for network analysis, two independent haplogroups (i.e., China and Japan) with higher levels of reticulation were identified. A similar topological structure was recognized using the single COII dataset to reduce the complexity of reticulation and achieve clear exhibition (Fig. 2a). The overall network had two independent star-like haplogroups (China and Japan) and several individuals from Taiwan showed nested structures within the haplogroup of China (Fig. 2a), which indicated a typical population expansion [46]. AMOVA revealed a significant genetic variance when populations were grouped by phylogenetic lineages (i.e., China and Japan, 80.13 %, *p* < 0.01), where approximately 18.75 % of the genetic variation was within populations and only 1.12 % was among populations within the group (Table 2). Mantel tests showed no significant IBD relationship between *Фst* and Euclidean distances in the two divergent lineages (Fig. 3), indicating that this species was not dispersal-limited and thus might possess a strong capacity for long-distance dispersal. Zones of genetic discontinuities identified by the Barrier 2.2 program showed a potential association of geographic barriers with genetic abruption (Additional file 6: Figure S3). When *K* = 1, the chief bold line first divided the entire sampling region into the China and Japan subregions, which reflected significant genetic/geographical isolation between them (Additional file 6: Figure S3a). When *K* = 2, the second bold line separated Taiwan from Mainland China, indicating that the population from Taiwan also had a certain degree of differentiation from Mainland China populations (Additional file 6: Figure S3b).

**Table 2:** Hierarchical analyses of molecular variance for *A. obscuriceps*

Genetic marker	Regional subdivisions	Source of variation	Variance explained	*P*	Fixation index
mtDNA	China & Japan groups	Among group	80.13	0.00	0.801
		Among populations within groups	1.12	0.00	0.056
		Within populations	18.75	0.00	0.812
nrDNA	China & Japan groups	Among group	83.50	0.00	0.835
		Among populations within groups	0.52	0.17	0.032
		Within populations	15.98	0.00	0.840

mtDNA, mitochondrial DNA; nrDNA, nuclear DNA

For the nuclear sequence data, 418 bp partial fragments of the ITS1 gene were successfully obtained from 178 individuals. A total of 10 unique haplotypes were derived from all individuals. The 10 polymorphic sites included 6 singleton variables and 4 parsimony informative sites. The nucleotide diversities and haplotype diversities ranged from 0 to 0.00292 (mean 0.00304) and from 0 to 0.711 (mean 0.664) respectively (Additional file 7: Table S4). Two haplogroups were also recognized in the MJ haplotype network (Fig. 2b), reflecting a significant genetic isolation between populations in China and Japan. The population in Taiwan included two haplotypes: one related to the ancestral haplotype of Mainland China, and the other a derivative haplotype (Fig. 2b). The star-like shapes in the network also indicated that *A. obscuriceps* experienced population expansion events (Fig. 2b). AMOVA indicated that the genetic variation was 83.50 % among groups (*p* < 0.01), 0.52 % among populations within groups, and 15.98 % within populations (Table 2). The large differentiation indicated that allele frequencies were different between the samples from China and Japan. Barrier analysis also showed that major differentiation occurred between the populations from China and Japan (*K* = 1) (Additional file 6: Figure S3c), and the population from Taiwan showed slight divergence from Mainland China (*K* = 4) (Additional file 6: Figure S3d).

### Coalescent inferences of demographic history

**Fig. 4:**
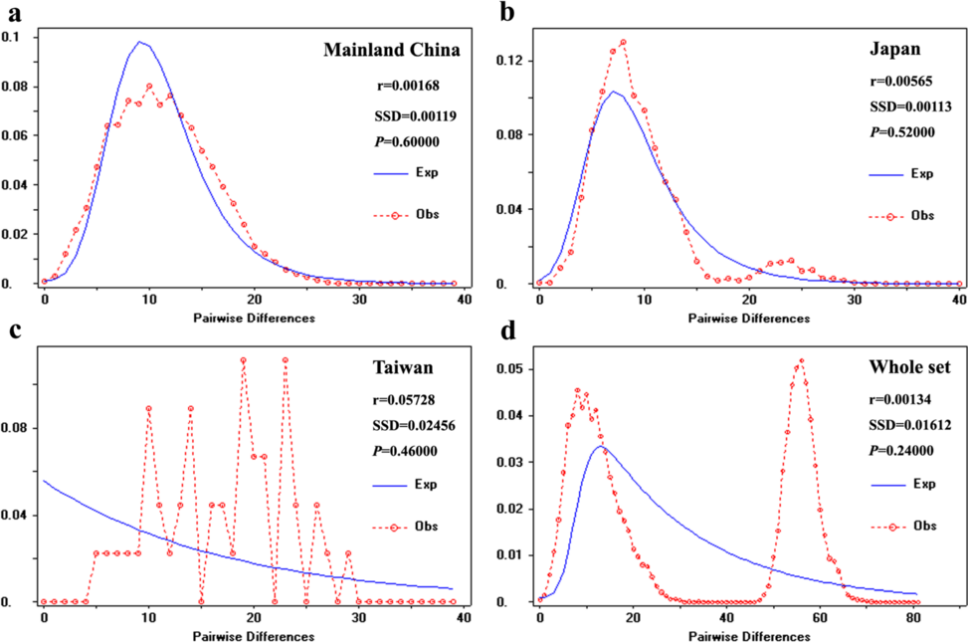
Mismatch distributions for both the subregional groups and the entire samples based on mitochondrial data. Curves representing the frequency of distribution pairwise differences: observed (Obs), expected (Exp). **a **Mainland China. **b** Japan. **c** Taiwan. **d** Whole set

Multimodal mismatch distributions are assumed to characterize old populations of constant size, whereas expanding populations are considered to be unimodal [47]. Mismatch distribution analyses revealed a multimodal distribution for the population from Taiwan (Fig. 4c), whereas a sudden expansion model with small and insignificant Harpending’s raggedness index was obtained in Mainland China, Japan, and the entire sample regions (*r* = 0.00134–0.00565) (Fig. 4a, b & d). The significantly negative values of Tajima’s *D*, Fu’s FS, and Fu and Li’s D* (Table 3) indicated a scenario of demographic expansion in Mainland China, Japan, and all sample populations. Combined results of neutrality tests, the nonsignificant parameters and the multimodal mismatch distributions indicated a model of relatively constant population size in the population from Taiwan. The result of EBSP revealed a relatively explicit demographic history for the three regions (i.e., Mainland China, Japan, and Taiwan) and the entire set (Additional file 8: Figure S4). The results showed that the population from Taiwan kept a nearly stable and small population size (Additional file 8: Figure S4c), corresponding to its narrow and limited distribution, whereas the populations in Mainland China, Japan and the entire region showed a constantly increasing population size over time after a period of low-efficiency population sizes (Additional file 8: Figure S4a, b, d). After transforming the units of the *x*-axes to years, the population expansion of *A. obscuriceps* appeared to begin approximately 0.14 Ma before present (BP).

**Table 3:** Nucleotide polymorphisms and Neutrality tests in defined groups and whole dataset based on mitochondrial DNA data

Parameter	Mainland China	Japan	Taiwan	Whole set
Sample size	121	57	10	188
*S*	179	135	51	277
*Nhap*	116	56	10	182
*Hd*	0.9989	0.9990	1.0000	0.9995
*π*	0.00707	0.00598	0.01072	0.01926
Tajima’s *D*	−2.24523**	−2.42838**	−0.28481	−1.30466
Fu’s Fs	−24.27842***	−24.66929***	−2.15008	−23.71411**
Fu and Li’s D*	−3.94409**	−3.45690**	−0.56710	−3.94421**

*S*, number of segregating sites; *NHap*, number of haplotypes; *Hd*, haplotype diversity; *π*, nucleotide diversity.*P < 0.05; **P < 0.02; ***P < 0.001

### Divergence time and migration rate

Simulation with IMa2 revealed unambiguous marginal posterior probability distributions of the demographic parameters. Divergence time was estimated to be 1.07 (95 % CI = 0.79–1.32) Ma between the population in China and that in Japan and 0.15 (95 % CI = 0.14–0.24) Ma between that in Mainland China and that in Taiwan. We also detected one statistically significant migration event of *A. obscuriceps* populations from Mainland China to Taiwan with *m* = 0.9995, which was greater than zero and thus indicated that gene flow occurred during or soon after the divergence event. Estimation of the divergence time by BEAST was presented in Figure S5 (Additional file 9: Figure S5).

### Paleoclimate niche modeling

**Fig. 5:**
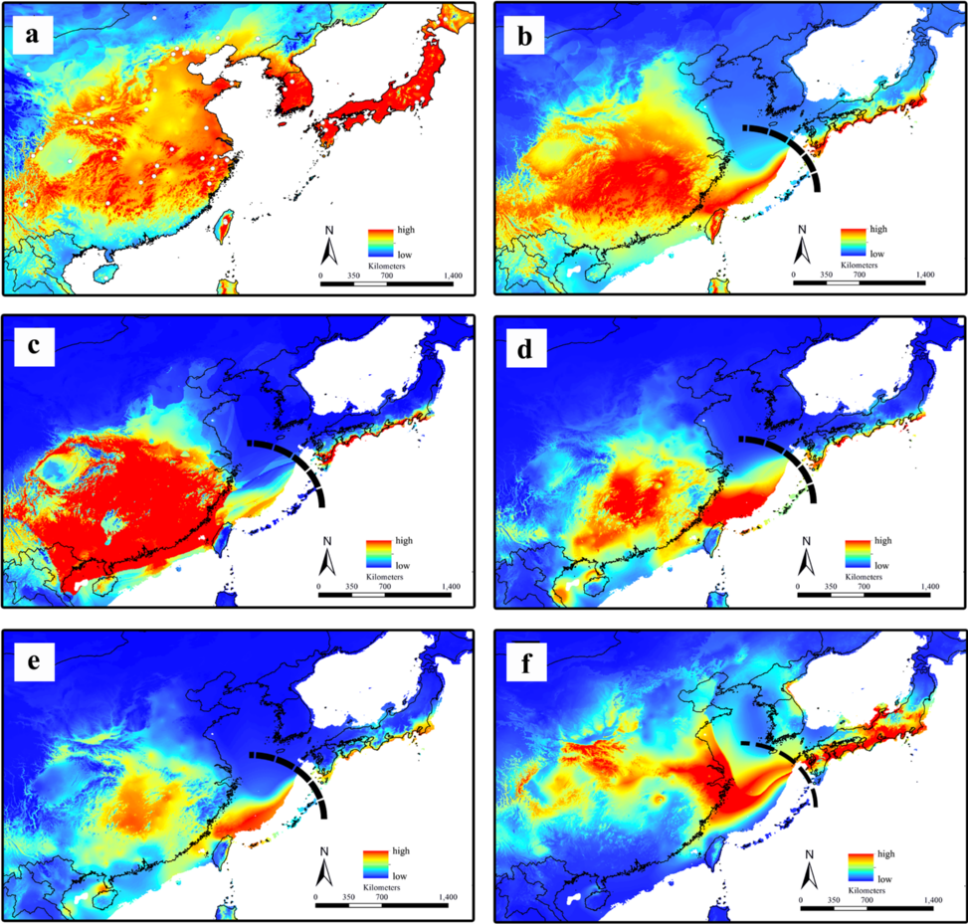
Modelled suitable areas of *A. obscuriceps* and related four host plants across ECS. **a** The current suitable areas of *A. obscuriceps*. **b** The LGM suitable areas of *A. obscuriceps*. **c** The LGM suitable areas of *Castanea mollissima*. **d** The LGM suitable areas of *Salix babylonica*. **e** The LGM suitable areas of *Salix matsudana*. **f** The LGM suitable areas of *Sorbaria sorbifolia*. LGM, the Last Glacial Maximum (21,000 – 18,000 year BP). Black bold lines represent habitat gap/barrier

The AUC of the ROC had values higher than 0.90 in all analyses, indicating a reasonable predictive model performance. Jackknife of regularized training gain for *A. obscuriceps* was shown in Figure S6 (Additional file 10: Figure S6). For *A. obscuriceps*, highly suitable areas were observed in the native range, which included most of East China, the Korean Peninsula and Japan (Fig. 5a). Integrating the current niche model into the simulated LGM climate conditions suggested that the potential range of the species contracted moderately toward the south. The highly suitable areas were mainly located in two segregated regions (Fig. 5b): the large-scale region distributed from southern China and extending to the continental shelf of the ECS across Taiwan and the narrow region located at the south coastal zone of Japan. The current potential areas of the four host plants of *A. obscuriceps* were also modeled according to their native areas. After projecting the current niche into the LGM historical climate condition, three of the 4 host plant species, *C. mollissima*, *Sa. babylonica* and *Sa. matsudana*, exhibited a relatively coincidental pattern of potential distribution in their native areas (Fig. 5c, d & e), which was similar to that of *A. obscuriceps* (Fig. 5b). The suitable LGM area of the host plant species *So. sorbifolia* showed slight differences compared with the other plants, indicating that suitable areas excluded Taiwan and expanded moderately toward the north in Japan (Fig. 5f). Notably, our LGM distribution model maps of *A. obscuriceps* and the four related host plants exhibited similar habitat gaps as geographical barriers on the exposed ECS continental shelf between China and Japan (Fig. 5b, c, d, e & f). Outside of the native range areas, highly suitable areas of *A. obscuriceps* identified by ENM included the northeastern areas along the Pacific coast, the eastern states in North America, Western Europe, areas around the Black Sea, South Eastern Australia, most of New Zealand, Central and Southern Africa, and New Guinea (Additional file 11: Figure S7). Areas in Southern Brazil and Chile and those along the Pacific coast of Columbia, Ecuador and Peru also showed high climate suitability (Additional file 11: Figure S7). Our niche model successfully identified the currently disjunct distribution pattern of *A. obscuriceps* on both sides of the Atlantic Ocean through comparisons with the actual invasion records, which indicated the predictive power of our model. In addition, the actual records of the four host plants probably matched the areas of potential invasion of *A. obscuriceps* outside the native areas (Additional file 12: Figure S8).

## Discussion

### Divergence time and underlying factors

The results of mitochondrial and nuclear DNA showed that *A. obscuriceps* comprised two main phylogenetic lineages, one from China and one from Japan, with reciprocally exclusive haplotypes that occupied distinct geographic distributions in China and Japan. The major genetic and geographical isolations achieved in our study were similar to several other species including the temperate plant *Kalopanax septemlobus* (Thunb.) Koidz., 1784 [48], the horseshoe bat *Rhinolophus ferrumequinum* (Schreber, 1774) [49], and the cotton pest *Adelphocoris suturalis* (Jakovlev, 1882) [50]. Based on the results of phylogenetic and haplotype network analyses, both sides of the ECS shared private haplotypes but did not share ancestral haplotypes, which suggested that divergence could have alternatively occurred long time ago and no substantial gene flow between the two regions were detectable since the divergence time, in accordance with the result of IMa2. Therefore, dispersal from China to Japan (or vice versa) through the hypothesized dispersal corridor (e.g., ECS land bridge) during the Pleistocene could have rarely occurred. However, the molecular results were inclined to disclose the more realistic situation that a former ancestor was extensively distributed in the pan-China-Japan region and was divided into two isolated groups because of the submergence of the ECS land bridge, as generally expected under a mode of vicariance/allopatric differentiation [51]. From late Miocene to Pleistocene, three periods of ECS land bridge formation and connection occurred between the Eurasian continent and the Japanese Islands at approximately 5.0–7.0 Ma, 1.3–2.0 Ma and 0.015–0.2 Ma respectively [52]. During these periods, most of the wide continental shelves of the ECS were exposed. Although the divergence time between the Chinese and Japanese lineages estimated by the IMa2 (i.e., 1.07 [0.79–1.32] Ma) varied in a relatively extensive range because of uncertainty in the mutation rate of mitochondrial DNA, our estimated divergence time and its HPD intervals approximately fell into the period of the “Ryukyu Coral Sea Stage” (0.2–1.3 Ma). During this period, the ECS sea level increased rapidly, and the dominantly exposed ECS land bridge area became entirely submerged [53]. Based on these paleo-data, we propose that the vicariance scenario led to divergent lineages of *A. obscuriceps* on both sides of the ECS. The ancestral status of *A. obscuriceps* was probably extensively distributed in East Asia, including the ECS land bridge in the early Pleistocene. At approximately 0.79–1.32 Ma, lineage differentiation between the population in China and that in Japan was driven by population contraction and extinction on the ECS land bridge because of sea level increase and land bridge submergence. However, we could not verify whether the ancestral status of *A. obscuriceps* was extensively distributed across East Asia in the early Pleistocene in this scenario. This phenomenon indicated that the cold and arid climates during the Plio-Miocene to early Pleistocene were less extensive than that during the middle and late Pleistocene [54]. Indeed, our LGM distribution model for *A. obscuriceps* showed that the potential range of the species contracted moderately in the Chinese region and sharply shrank toward the south coastal zone in the Japanese region. This finding suggested that *A. obscuriceps* was relatively sensitive to the low temperature in the northern regions during the LGM. Therefore, the Plio-Miocene to early Pleistocene environment might have created more favorable conditions for the ancestral status of *A. obscuriceps* to occupy relatively extensive and continuous regions in East Asia.

The results of phylogenetic and haplotype network analyses showed that several individuals from Taiwan were nested structures within the haplogroups of China, which indicated that a relatively shallow divergence occurred between the populations in Mainland China and that in Taiwan. The shared ancestral haplotypes showed that recent gene flow also existed between these two regions. The divergence time (i.e., 0.15 [0.10–0.22] Ma) between Mainland China and Taiwan approximated the penultimate glaciation period (0.13–0.30 Ma) [55], which was located at the transition time from the mid-Pleistocene to the late Pleistocene. The earth, particularly the Northern Hemisphere, experienced a sudden climate shift during the period called the “mid-Pleistocene revolution” [56]. The mid-Pleistocene climate revolution was characterized by a gradual change in the dominant climate periodicity from 40 ka to 100 ka centered on the marine isotope stage 23/22 boundary at approximately 0.9 million years [57]. This sudden shift in climate cycles resulted in increasing durations of cold periods, followed by fluctuation of the East Asian monsoon, which had profound effects on vegetation and creature responses [58]. Therefore, we speculate that the cold environment during the transition from the mid-Pleistocene to late Pleistocene induced the differentiation of *A. obscuriceps* populations between Mainland China and Taiwan.

### Inferences of the historical demography of *A. obscuriceps* in the native range

Following the inferred sundering in the early–middle Pleistocene, populations of *A. obscuriceps* experienced different demographic histories in Mainland China, Japan, and Taiwan. For the regions in Mainland China and Japan, the observed pairwise differences of mtDNA haplotypes fit the sudden expansion model particularly well (Fig. 4a & b), producing significantly negative neutrality test statistics (Table 3). This finding provides strong evidence of a recent demographic expansion in these two regions, which is also in line with the results of EBSP (Additional file 8: Figure S4a, b). Population expansion occurred approximately 0.14 Ma years ago and subsequently sustained a steady growth with the increase in population size over time, which coincided with the Last Interglacial (LIG) (0.12–0.14 Ma BP). A direct link between range expansion and the beginning of the warm climate seems plausible. Temperatures during the LIG were approximately 2–5 °C warmer than those in the present ECS region [48]. Thus, the warm and moist East Asian summer monsoons intensified [59], allowing the persistence of vegetation similar to that observed today [60], which consequently might also lead to an increase in suitable habitats for *A. obscuriceps* throughout the warmer periods of the LIG. During the LGM period, the ice sheets did not cover most of East Asia and the glacial cycle effects were less dramatic [61]. Thus, the mild climate relative to Europe and North America might have also mitigated the demographic stresses on *A. obscuriceps*. Maintenance of the large effect of population size during cooler periods might be also partially attributed to the population genetic structure. Theoretically, although several habitats were lost and fragmented because of the cold glacial period, the census size of populations may still be able to highly inflate as a result of frequent migration events among subpopulations due to strong dispersal ability [62]. Our results of the reticular haplotype network and nonsignificant IBD parameters indicate that this species possesses relatively strong dispersal ability for frequent gene flow, which conforms to the theoretical description. In this study, we proposed that, if *A. obscuriceps* was sheltered in multiple refugia during the LGM, then frequent migration among populations at all geographic distances might have occurred through its strong dispersal ability. Such continuous gene flow could have made *A. obscuriceps* less sensitive to habitat reduction and fragmentation driven by climate changes.

In contrast to Mainland China and Japan, the population in Taiwan has a relatively small size, which is consistent with the result of EBSP (Additional file 8: Figure S4c). The multimodal mismatch distributions, together with the nonsignificant parameter statistics (Fig. 4c; Table 3), indicate a long-term persistence of the Taiwan population in separate refugia without significant growth [29]. The only significant migration detected by the IMa2 was from Mainland China to Taiwan. This recent gene flow might have mitigated the divergence between the Mainland China and Taiwan populations, which also corresponded to the ancestral haplotype networks (Fig. 2). The lack of local ancestral haplotypes and the large number of private haplotypes, together with permanently small population sizes, indicate that *A. obscuriceps* in Taiwan might have experienced more severe extinctions and/or stronger genetic drift.

### Host plant habitat requirement as the key to *A. obscuriceps* colonization

Many factors influence the establishment of a nonindigenous species in a community, including physiological flexibility (e.g., dispersal ability and habitat requirement) [63]. The ENM can identify areas of matching climate space occupied by the invasive species in the native area. Prior to inferring potential areas, the ENM seeks to identify suitable climate spaces for species, without considering dispersal ability [40]. The Mantel tests did not reveal significant IBD patterns for *A. obscuriceps* (Fig. 3), indicating a strong dispersal ability, high gene flow at all geographic distances, and minimal genetic drift. The results of haplotype networks and AMOVA (Fig. 2; Table 2) also indicate complex and higher levels of reticulation within Chinese and Japanese lineages, which are characterized by higher gene flow among populations within a group at all geographic distances. Based on these results, the small and winged insect *A. obscuriceps* was likely to be a strong flier with presumably strong capacity for long-distance dispersal. The ENM prediction results showed that suitable habitats of this species were distributed in the partial continental shelves of the ECS and the south coastal zone of Japan, but with a short disjunct distance between them (Fig. 5b). Depending on its strong dispersal ability, *A. obscuriceps* could be expected to generate gene flow between these two regions. However, this standpoint was rejected because of the significantly divergent lineages between the populations in China and Japan (Fig. 2). Instances of this type of situation were observed in the steppe bird species *Tetrax tetrax* (Linnaeus, 1758) [64] and two grasshopper species *Hesperotettix viridis* (Thomas, 1872) and *Ceracris kiangsu* Tsai, 1929 [63, 65]. This puzzling issue might be sorted out by focusing on the habitat requirement of this species. The highly suitable areas during the LGM predicted by the ENM of the four host plants showed a similar distribution pattern, which was consistent with the predicted area of *A. obscuriceps*: similar unsuitable area gaps existed as the geographical barrier between the ECS continental shelf and Japanese regions (Fig. 5c, d, e & f). Thus, we speculated that the discontinuous distribution of the four host plants shaped a habitat filter that probably impeded the gene flow of *A. obscuriceps* between the populations in China and Japan. From another point of view, this hypothesis could be supported by evidence from populations in Mainland China and Taiwan. Individuals of Taiwan were partially nested structures within haplogroups of China (Fig. 2), indicating a recent limited gene flow that probably occurred after the divergence event. The LGM prediction showed that the suitable habitat of *A. obscuriceps* occupying a continuous range covered the southeast coastal areas of Mainland China and Taiwan across the Taiwan Strait, which shaped a large and intact refuge. Coincidentally, the same distribution patterns were detected in the LGM prediction of the three host plants *C. mollissima*, *Sa. babylonica* and *Sa. matsudana* (Fig. 5c, d & e), indicating continuous suitable areas across the Taiwan Strait. Thus, the continuous distribution of these host plants possibly shaped a habitat corridor, which was coupled with a moderate dispersal ability and promoted gene flow between the populations in Mainland China and Taiwan.

## Conclusions

Phylogeography coupled with ENM revealed the Pleistocene history of the invasive species *A. obscuriceps* in its native range. Molecular data revealed a significant genetic differentiation between the populations in China and Japan. Divergence time approximately coincided with the period of sea level increase, and recent gene flow was not detected by the IMa2 analysis. We determined that the vicariance scenario led to divergent lineages of *A. obscuriceps* on both sides of the ECS. No significant IBD relationship was observed between *Фst* and the Euclidean distances in the Mantel tests, which is consistent with the hypothesis that this species has a good dispersal ability. Our LGM niche modeling of the four related host plants exhibited similar habitat gaps on the exposed ECS continental shelf between China and Japan, but showed continuous distribution across the Taiwan Strait. We provided a successful example of uncovering the genetic structure and underlying factors of an invasive insect across the ECS. The findings of our study will also shed light on the potential role of habitat requirement in the process of biological invasion.

## Additional files

Additional file 1: Table S1. PCR primer sequences used to amplify mitochondrial and nuclear fragments. (DOC 33 kb) Additional file 2: Table S2. Pearson’s correlation coefficient (*R*) for the 7 bioclimatic variables. (DOC 36 kb) Additional file 3: Table S3. Discovery of *Amphiareus obscuriceps* in different countries and related reference. (DOC 44 kb) Additional file 4: Figure S1. Phylogenetic Bayesian tree analysis of all 182 haloptypes from mitochondrial markers (COI + COII + CytB). Branches above 50 % Bayesian posterior probabilities are shown in number. (DOC 53 kb) Additional file 5: Figure S2. Phylogenetic ML tree analysis of all 182 haloptypes from mitochondrial markers (COI + COII + CytB). ML bootstrap percentages above 50 % bootstrap support are shown in number. (DOC 41 kb) Additional file 6: Figure S3. Zones of genetic discontinuities. Barriers were retained under the majority-rule criteria according to their importance. (a & b) Mitochondrial data. (c & d) Nuclear data. (DOC 1636 kb) Additional file 7: Table S4. Nucleotide polymorphisms in each geographic population. *S*, number of segregating sites; *NHap*, number of haplotypes; *Hd*, haplotype diversity; *π*, nucleotide diversity. (DOC 60 kb) Additional file 8: Figure S4. Extended Bayesian Skyline Plots (EBSP), inferred from mitochondrial and nuclear DNA sequence variations (COI, CytB, ITS1) depicting changes in effective population size (*Ne*) as a function of time for Mainland China (a), Japan (b), Taiwan (c), and the whole set (d). The thick solid black line marks the estimated medians, and the area delimited by the upper and lower grey lines represents the HPD 95 % confidence intervals for *Ne*. (DOC 117 kb) Additional file 9: Figure S5. Divergence time among the three mitochondrial haplogroups (Mainland China, Japan and Taiwan) calculated by BEAST. The area delimited by blue lines represents the HPD 95 % confidence intervals. (DOC 58 kb) Additional file 10: Figure S6. Jackknife of regularized training gain for *A. obscuriceps*. (DOC 36 kb) Additional file 11: Figure S7. Niche model based on native records of *A. obscuriceps* and those transferred worldwide using Maxent. Dark red color represents higher suitability, while dark blue indicates lower suitability. Black and red dots represent the native and invasive records respectively. (DOC 111 kb) Additional file 12: Figure S8. Coordinates of four host plants’ positions on the map of predicted invasive areas of *A. obscuriceps*. Dark red color represents higher suitability, while dark blue indicates lower suitability. Black and red dots represent the native and invasive records respectively. (a) *Castanea mollissima*; (b) *Salix babylonica*; (c) *Salix matsudana*; (d) *Sorbaria sorbifolia*. (DOC 1211 kb)

## Abbreviations

AIC: Akaike information criterion
AMOVA: Analysis of molecular variance
AUC: The areas under the curve
BGI: Beijing Genomics Institute
BI: Bayesian inference
BP: Before present
COI: Cytochrome c oxidase subunit I
COII: Cytochrome c oxidase subunit II
CytB: Cytochrome b
EBSP: Extended Bayesian skyline plots
ECS: The East China Sea
ENM: Ecological niche modeling
ESS: Effective sample size
GBIF: Global Biodiversity Information Facility
IBDWS: Isolation by Distance Web Service
IM: Isolation-with-migration
ITS1: Internal transcribed spacer 1
LGM: Last Glacial Maximum
LIG: Last Interglacial
MCMC: Markov Chain Monte Carlo
ML: Maximum likelihood
ROC: Receiver operating characteristic
SJR: Sino-Japanese region.
