# Supplementary material for: Pleistocene sea level fluctuation and host plant habitat requirement influenced the historical phylogeography of the invasive species Amphiareus obscuriceps (Hemiptera: Anthocoridae) in its native range
Source: BMC Evol Biol. 2016 Aug 31;16(1):174. doi: 10.1186/s12862-016-0748-3 (PMC5007872; doi:10.1186/s12862-016-0748-3)
Supplement: Additional file 1: Table S1. — PCR primer sequences used to amplify mitochondrial and nuclear fragments. (DOC 33 kb) [file 12862_2016_748_MOESM1_ESM.doc]

**Additional file 1: Table S1.** PCR primer sequences used to amplify mitochondrial and nuclear fragments.

| **Gene region** | **Primer name** | **Sequence (5′–3′)** |
| --- | --- | --- |
| **COI** | Yt1f | AAACTATTAACCTTCAAAG |
|  | HCO2198 | TAAACTTCAGGGTGACCAAAAAATCA |
| **COII** | COII-1 | AATATGGCAGATTAGTGCA |
|  | COII-2 | GCTCCACAAATTTCTGAGCA |
| **CytB** | P6 | TATGTTCTTCCCTGAGGACAAATATC |
|  | P7 | ATTACACCTCCTAATTTATTAGGAAT |
| **ITS1** | AA5 | CCGATTGAATGATTTAGTGAGGTCT |
|  | R58sg | GCCTTTGGGATTCAGTGCT |
