# Supplementary material for: Pleistocene sea level fluctuation and host plant habitat requirement influenced the historical phylogeography of the invasive species Amphiareus obscuriceps (Hemiptera: Anthocoridae) in its native range
Source: BMC Evol Biol. 2016 Aug 31;16(1):174. doi: 10.1186/s12862-016-0748-3 (PMC5007872; doi:10.1186/s12862-016-0748-3)
Supplement: Additional file 2: Table S2. — Pearson’s correlation coefficient (R) for the 7 bioclimatic variables. (DOC 36 kb) [file 12862_2016_748_MOESM2_ESM.doc]

**Additional file 2: Table S2. Pearson’s correlation coefficient (*R*) for the 7 bioclimatic variables.**

|  | BIO 1 | BIO 2 | BIO 5 | BIO 6 | BIO 12 | BIO 13 | BIO 14 |
| --- | --- | --- | --- | --- | --- | --- | --- |
| BIO 1 |  |  |  |  |  |  |  |
| BIO 2 | -0.496 |  |  |  |  |  |  |
| BIO 5 | 0.764 | -0.033 |  |  |  |  |  |
| BIO 6 | 0.774 | -0.609 | 0.569 |  |  |  |  |
| BIO 12 | 0.714 | -0.289 | 0.403 | 0.766 |  |  |  |
| BIO 13 | 0.723 | -0.271 | 0.436 | 0.739 | 0.789 |  |  |
| BIO 14 | -0.345 | -0.106 | 0.207 | 0.416 | 0.714 | 0.477 |  |
