# Supplementary material for: Pleistocene sea level fluctuation and host plant habitat requirement influenced the historical phylogeography of the invasive species Amphiareus obscuriceps (Hemiptera: Anthocoridae) in its native range
Source: BMC Evol Biol. 2016 Aug 31;16(1):174. doi: 10.1186/s12862-016-0748-3 (PMC5007872; doi:10.1186/s12862-016-0748-3)
Supplement: Additional file 3: Table S3. — Discovery of Amphiareus obscuriceps in different countries and related reference. (DOC 44 kb) [file 12862_2016_748_MOESM3_ESM.doc]

**Additional file 3: Table S3.** Discovery of *Amphiareus obscuriceps* in different countries and related reference.

| Region | Year when first reported | Reference |
| --- | --- | --- |
| Canada (Ontario) | 1978 | Kelton 1978 |
| US (Maryland) | 1983 | Henry 2008 |
| Bulgaria | 1987 | Péricart and Stehlík 1998 |
| Hungary | 1989 | Aukema 1990a |
| Belarus | 1992 | Rosenzweig 1995 |
| Czech Republic | 1994 | Jindra and Kabiček 2001 |
| Italy | 1995 | Bacchi and Rizzotti-Vlach 2000 |
| Austria | 1998 | Friess 2000 |
| Slovakia | 2000 | Kment *et al*. 2003 |
| Germany | 2001 | Simon 2002 |
| Finland | 2003 | Albrecht *et al*. 2003 |
| Netherlands | 2003 | Aukema *et al*. 2005a |
| Estonia | 2004 | Selin 2004 |
| Belgium | 2007 | Aukema *et al*. 2007 |

Reference:

Albrecht A, Söderman G, Rinne V, Mattila K, Mannerkoski I, Karjalainen S, et al. New and interesting finds of Hemiptera in Finland. Sahlbergia. 2003;8:64-78.

Aukema B. Additional data on the Heteroptera fauna of the Kiskunság National Park. Folia Entomologica Hungarica. 1990a;51:5-16.

Aukema B, Bruers J, Viskens G. Nieuwe en zeldzame Belgische wantsen II (Hemiptera: Heteroptera). Bulletin van de Koninklijke Belgische Vereniging voor Entomologie. 2007;143:83-91.

Aukema B, Bos F, Hermes D, Zeinstra P. Nieuwe en interessante nederlandse wantsen II, met een geactu-aliseerde naamlijst (Hemiptera: Heteroptera). Nederlandse Faunistische Mededelingen. 2005a;23:37-76.

Bacchi I, Rizzotti Vlach M. *Amphiareus obscuriceps* in Italia: note morfologiche, ecologiche e corologiche (Heteroptera, Anthocoridae). Bollettino della Societa Entomologica Italiana. 2000;132:99-103.

Friess T. Libellen (Odonata) und Wanzen (Heteroptera) aus dem Naturschutzgebiet "Gut Walterskirchen" am Wörthersee. Carinthia II.2000;190./110.:517-30.

Henry TJ, Wheeler Jr AG, Steiner Jr WE. First North American records of *Amphiareus obscuriceps* (Poppius) (Hemiptera: Heteroptera: Anthocoridae), with a discussion of dead-leaf microhabitats. Proc Entomol Soc Wash. 2008;110:402-16.

Jindra Z, Kabiček J. Faunistic records from the Czech Republic – 130. Klapalekiana. 2001;37:124.

Kelton LA. The Anthocoridae of Canadaand Alaska. The Insects and Arachnids of Canada. Part 4. Agriculture Canada Research Publication. 1978;1639:101.

Kment P, Bryja J, Hradil K, Jindra Z, Baňař P. New and interesting records of true bugs (Heteroptera) from the Czech Republic and Slovakia II. Klapalekiana. 2003;39:257-306.

Péricart J, Stehlík JL. *Amphiareus obscuriceps* (Popp.) in the Czech Republic and in the Balkan Peninsula (Heteroptera: Anthocoridae). Acta Musei Moraviae, Scientiae biologicae. 1998;83:217-8.

Rosenzweig VE. Addition to the fauna of the Heteroptera of Belarus. Trudy Zoologicheskogo Muzeya Belorusskogo Gosudarstvennogo Universiteta. 1995;1:267-71.

Selin A. Uusi lutikalisi (Heteroptera) eesti faunas. Lepinfo. 2004;15:57-8.

Simon H. Erstes vorläufiges Verzeichnis der Wanzen (Insecta: Heteroptera) in Rheinland-Pfalz. Fauna und Flora Rheinland-Pfalz. 2002;9:1379-420.
