# Supplementary material for: Pleistocene sea level fluctuation and host plant habitat requirement influenced the historical phylogeography of the invasive species Amphiareus obscuriceps (Hemiptera: Anthocoridae) in its native range
Source: BMC Evol Biol. 2016 Aug 31;16(1):174. doi: 10.1186/s12862-016-0748-3 (PMC5007872; doi:10.1186/s12862-016-0748-3)
Supplement: Additional file 5: Figure S2. — Phylogenetic ML tree analysis of all 182 haloptypes from mitochondrial markers (COI + COII + CytB). ML bootstrap percentages above 50 % bootstrap support are shown in number. (DOC 41 kb) [file 12862_2016_748_MOESM5_ESM.doc]

**Additional file 5: Figure S2.** Phylogenetic ML tree analysis of all 182 haloptypes from mitochondrial markers (COI+COII+CytB). ML bootstrap percentages above 50% bootstrap support are shown in number.

**
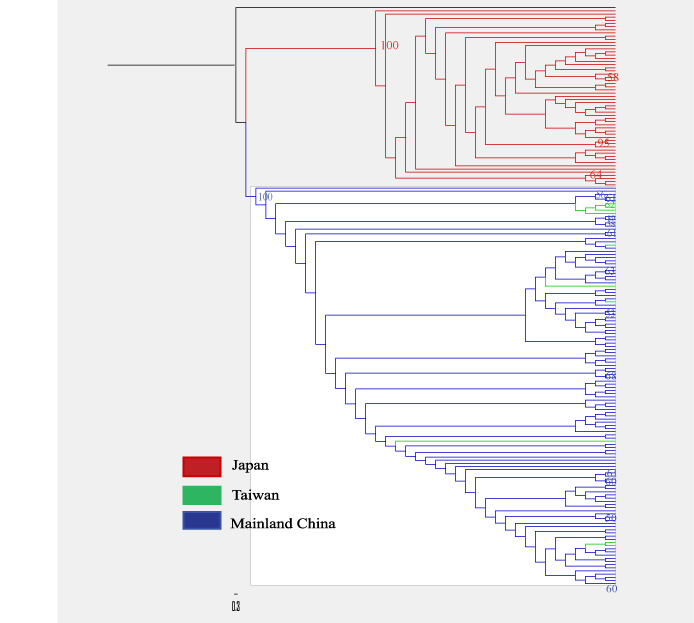
**
