# Supplementary material for: Pleistocene sea level fluctuation and host plant habitat requirement influenced the historical phylogeography of the invasive species Amphiareus obscuriceps (Hemiptera: Anthocoridae) in its native range
Source: BMC Evol Biol. 2016 Aug 31;16(1):174. doi: 10.1186/s12862-016-0748-3 (PMC5007872; doi:10.1186/s12862-016-0748-3)

**Additional file 6: Figure S3.** Zones of genetic discontinuities. Barriers were retained under the majority-rule criteria according to their importance. (a & b) Mitochondrial data. (c & d) Nuclear data.


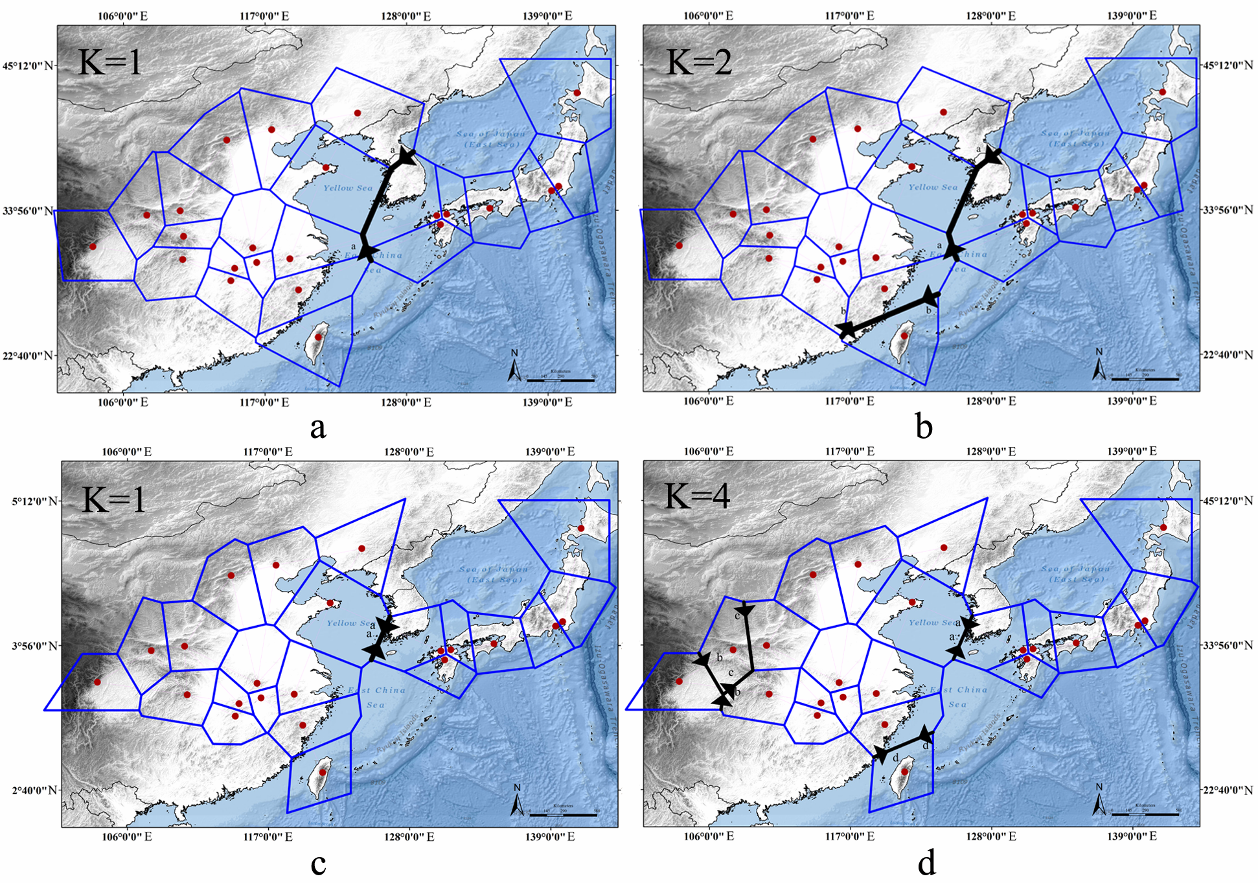

Supplement: Additional file 6: Figure S3. — Zones of genetic discontinuities. Barriers were retained under the majority-rule criteria according to their importance. (a & b) Mitochondrial data. (c & d) Nuclear data. (DOC 1636 kb) [file 12862_2016_748_MOESM6_ESM.doc]
