# Supplementary material for: Pleistocene sea level fluctuation and host plant habitat requirement influenced the historical phylogeography of the invasive species Amphiareus obscuriceps (Hemiptera: Anthocoridae) in its native range
Source: BMC Evol Biol. 2016 Aug 31;16(1):174. doi: 10.1186/s12862-016-0748-3 (PMC5007872; doi:10.1186/s12862-016-0748-3)
Supplement: Additional file 7: Table S4. — Nucleotide polymorphisms in each geographic population. S, number of segregating sites; NHap, number of haplotypes; Hd, haplotype diversity; π, nucleotide diversity. (DOC 60 kb) [file 12862_2016_748_MOESM7_ESM.doc]

**Additional file 7: Table S4.** Nucleotide polymorphisms in each geographic population. *S*, number of segregating sites; *NHap*, number of haplotypes; *Hd*, haplotype diversity; *π*, nucleotide diversity.

| **ITS1** | **Lat.** | **Long.** | **Sample size** | ***S*** | ***Nhap*** | ***Hd*** | ***π*** |
| --- | --- | --- | --- | --- | --- | --- | --- |
| **Mainland China** |  |  |  |  |  |  |  |
| AH | 30°58'57" | 116°4'49" | 10 | 1 | 2 | 0.200 | 0.00048 |
| CQ | 29°50'16" | 106°23'48" | 9 | 1 | 2 | 0.222 | 0.00053 |
| FP | 33°31'51" | 107°49'45" | 10 | 2 | 3 | 0.600 | 0.00175 |
| HBTS | 29°23'57" | 114°40'51" | 1 | – | 1 | – | – |
| HBWF | 30°4'40" | 110°37'33" | 1 | – | 1 | – | – |
| JX | 28°25'9" | 114°23'3" | 10 | 1 | 2 | 0.467 | 0.00112 |
| LN | 41°29'51" | 124°14'56" | 12 | 1 | 2 | 0.485 | 0.00116 |
| SC | 31°4'5" | 103°37'49" | 10 | 3 | 4 | 0.711 | 0.00292 |
| SD | 37°14'34" | 121°46'41" | 8 | 1 | 2 | 0.250 | 0.00060 |
| SN | 33°52'29" | 110°25'54" | 9 | 2 | 3 | 0.417 | 0.00106 |
| SX | 39°23'2" | 114°3'40" | 12 | 1 | 2 | 0.485 | 0.00116 |
| TJ | 40°11'16" | 117°33'22" | 5 | 1 | 2 | 0.600 | 0.00144 |
| ZJ | 30°7'54" | 118°59'4" | 15 | 1 | 2 | 0.476 | 0.00114 |
| ZJTS | 27°42'29" | 119°39'4" | 8 | 1 | 2 | 0.536 | 0.00128 |
| **Japan** |  |  |  |  |  |  |  |
| JC | 35°47'15" | 139°54'11" | 8 | 1 | 2 | 0.250 | 0.00060 |
| JF | 33°29'58" | 130°25'19" | 9 | 0 | 1 | – | – |
| JH | 43°3'44" | 141°21'16" | 6 | 0 | 1 | – | – |
| JK | 32°48'11" | 130°42'28" | 8 | 1 | 2 | 0.250 | 0.00060 |
| JO | 33°35'54" | 131°11'18" | 10 | 0 | 1 | – | – |
| JP | 35°26'35" | 139°21'45" | 3 | 0 | 1 | – | – |
| JT | 34°4'13" | 134°33'17" | 10 | 0 | 1 | – | – |
| **Taiwan** |  |  |  |  |  |  |  |
| TW | 24°1'22" | 121°11'14" | 4 | 1 | 2 | 0.500 | 0.00120 |
