# Supplementary material for: Pleistocene sea level fluctuation and host plant habitat requirement influenced the historical phylogeography of the invasive species Amphiareus obscuriceps (Hemiptera: Anthocoridae) in its native range
Source: BMC Evol Biol. 2016 Aug 31;16(1):174. doi: 10.1186/s12862-016-0748-3 (PMC5007872; doi:10.1186/s12862-016-0748-3)
Supplement: Additional file 8: Figure S4. — Extended Bayesian Skyline Plots (EBSP), inferred from mitochondrial and nuclear DNA sequence variations (COI, CytB, ITS1) depicting changes in effective population size (Ne) as a function of time for Mainland China (a), Japan (b), Taiwan (c), and the whole set (d). The thick solid black line marks the estimated medians, and the area delimited by the upper and lower grey lines represents the HPD 95 % confidence intervals for Ne. (DOC 117 kb) [file 12862_2016_748_MOESM8_ESM.doc]

**Additional file 8: Figure S4.** Extended Bayesian Skyline Plots (EBSP), inferred from mitochondrial and nuclear DNA sequence variation (COI, CytB, ITS1) depicting changes in effective population size (*Ne*) as a function of time for Mainland China (a), Japan (b), Taiwan (c), and the whole set (d). The thick solid black line marks the estimated medians, and the area delimited by the upper and lower grey lines represents the HPD 95% confidence intervals for *Ne*.

**
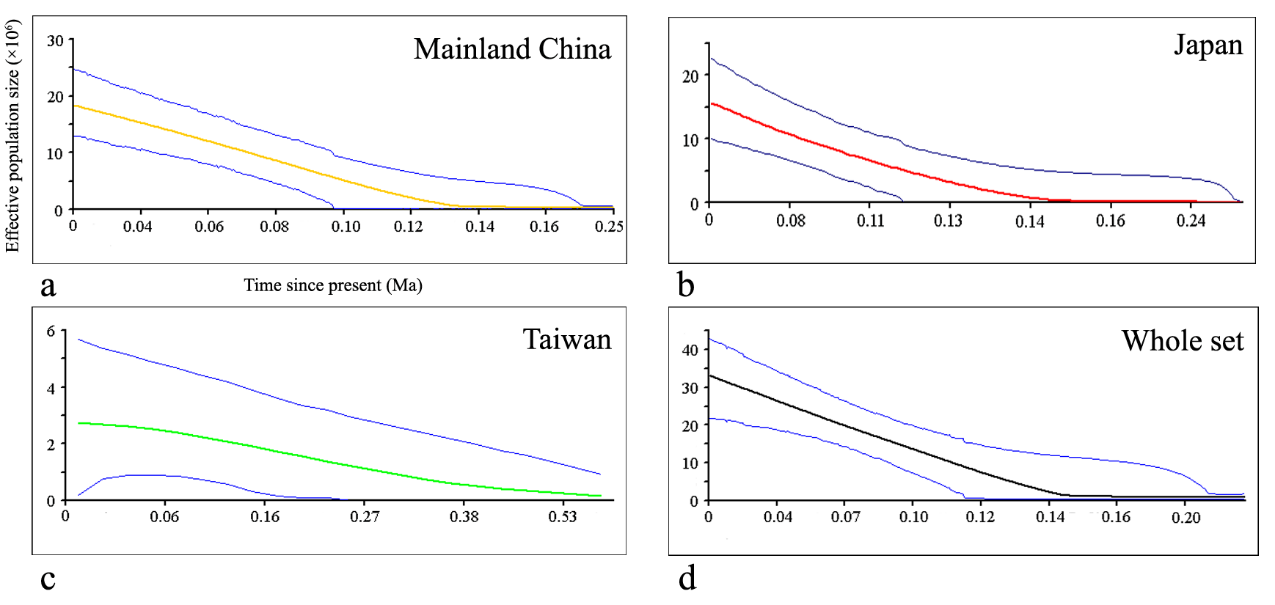
**
