# Supplementary material for: Pleistocene sea level fluctuation and host plant habitat requirement influenced the historical phylogeography of the invasive species Amphiareus obscuriceps (Hemiptera: Anthocoridae) in its native range
Source: BMC Evol Biol. 2016 Aug 31;16(1):174. doi: 10.1186/s12862-016-0748-3 (PMC5007872; doi:10.1186/s12862-016-0748-3)
Supplement: Additional file 9: Figure S5. — Divergence time among the three mitochondrial haplogroups (Mainland China, Japan and Taiwan) calculated by BEAST. The area delimited by blue lines represents the HPD 95 % confidence intervals. (DOC 58 kb) [file 12862_2016_748_MOESM9_ESM.doc]

**Additional file 9: Figure S5.** Divergence time among the three mitochondrial haplogroups (Mainland China, Japan and Taiwan) calculated by BEAST. The area delimited by blue lines represents the HPD 95% confidence intervals.

**
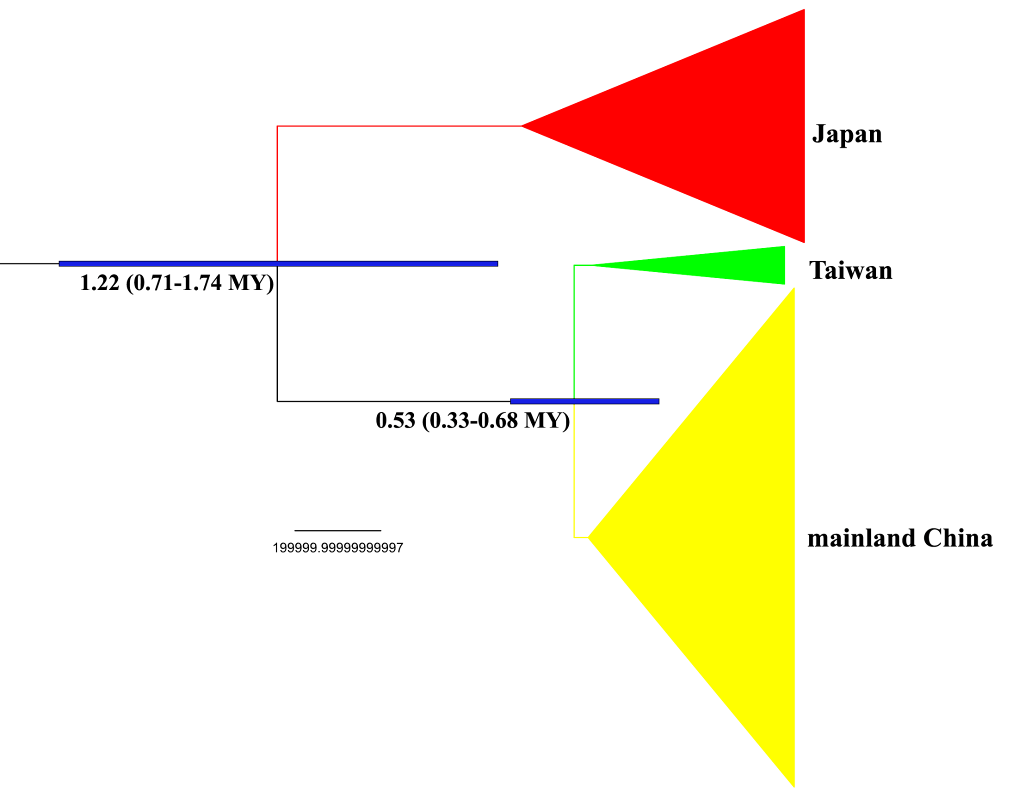
**
