# Supplementary figures and images for: Pleistocene sea level fluctuation and host plant habitat requirement influenced the historical phylogeography of the invasive species Amphiareus obscuriceps (Hemiptera: Anthocoridae) in its native range
Source: BMC Evol Biol. 2016 Aug 31;16(1):174. doi: 10.1186/s12862-016-0748-3 (PMC5007872; doi:10.1186/s12862-016-0748-3)

**Additional file 10: Figure S6.** Jackknife of regularized training gain for *A. obscuriceps*.


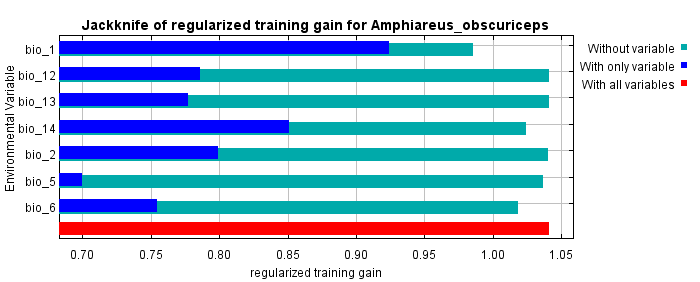

Supplement: Additional file 10: Figure S6. — Jackknife of regularized training gain for A. obscuriceps. (DOC 36 kb) [file 12862_2016_748_MOESM10_ESM.doc]
