# Supplementary material for: Pleistocene sea level fluctuation and host plant habitat requirement influenced the historical phylogeography of the invasive species Amphiareus obscuriceps (Hemiptera: Anthocoridae) in its native range
Source: BMC Evol Biol. 2016 Aug 31;16(1):174. doi: 10.1186/s12862-016-0748-3 (PMC5007872; doi:10.1186/s12862-016-0748-3)

**Additional file 11: Figure S7.** Niche model based on native records of *A. obscuriceps* and those transferred worldwide using Maxent. Dark red color represents higher suitability, while dark blue indicates lower suitability. Black and red dots represent the native and invasive records respectively.


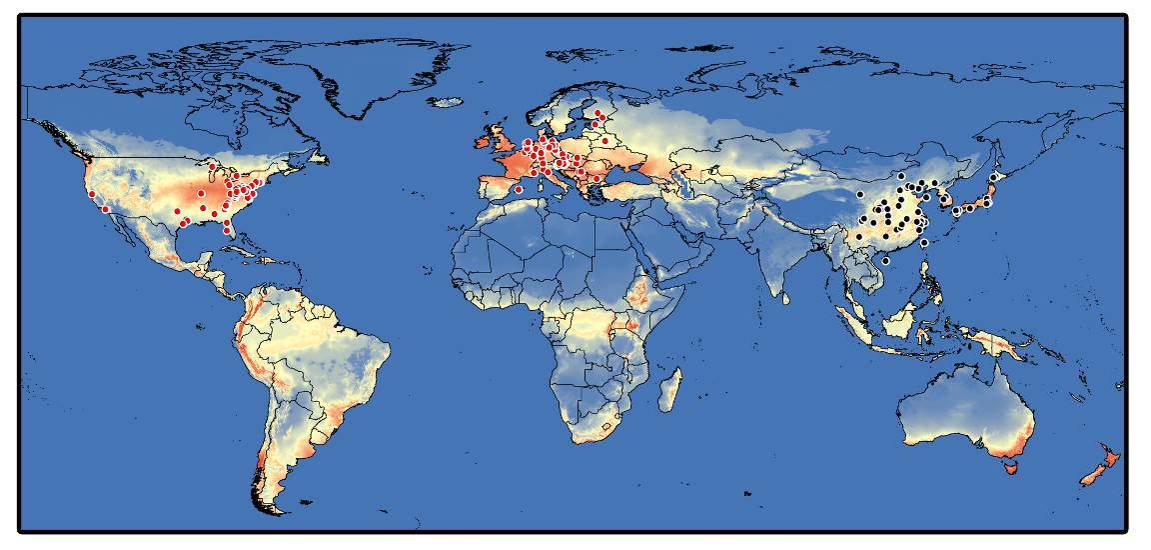

Supplement: Additional file 11: Figure S7. — Niche model based on native records of A. obscuriceps and those transferred worldwide using Maxent. Dark red color represents higher suitability, while dark blue indicates lower suitability. Black and red dots represent the native and invasive records respectively. (DOC 111 kb) [file 12862_2016_748_MOESM11_ESM.doc]
