# Supplementary material for: Pleistocene sea level fluctuation and host plant habitat requirement influenced the historical phylogeography of the invasive species Amphiareus obscuriceps (Hemiptera: Anthocoridae) in its native range
Source: BMC Evol Biol. 2016 Aug 31;16(1):174. doi: 10.1186/s12862-016-0748-3 (PMC5007872; doi:10.1186/s12862-016-0748-3)

**Additional file 12: Figure S8.** Coordinates of four host plants’ positions on the map of predicted invasive areas of *A. obscuriceps*. Dark red color represents higher suitability, while dark blue indicates lower suitability. Black and red dots represent the native and invasive records respectively. (a) *Castanea mollissima*; (b) *Salix babylonica*; (c) *Salix matsudana*; (d) *Sorbaria sorbifolia*.


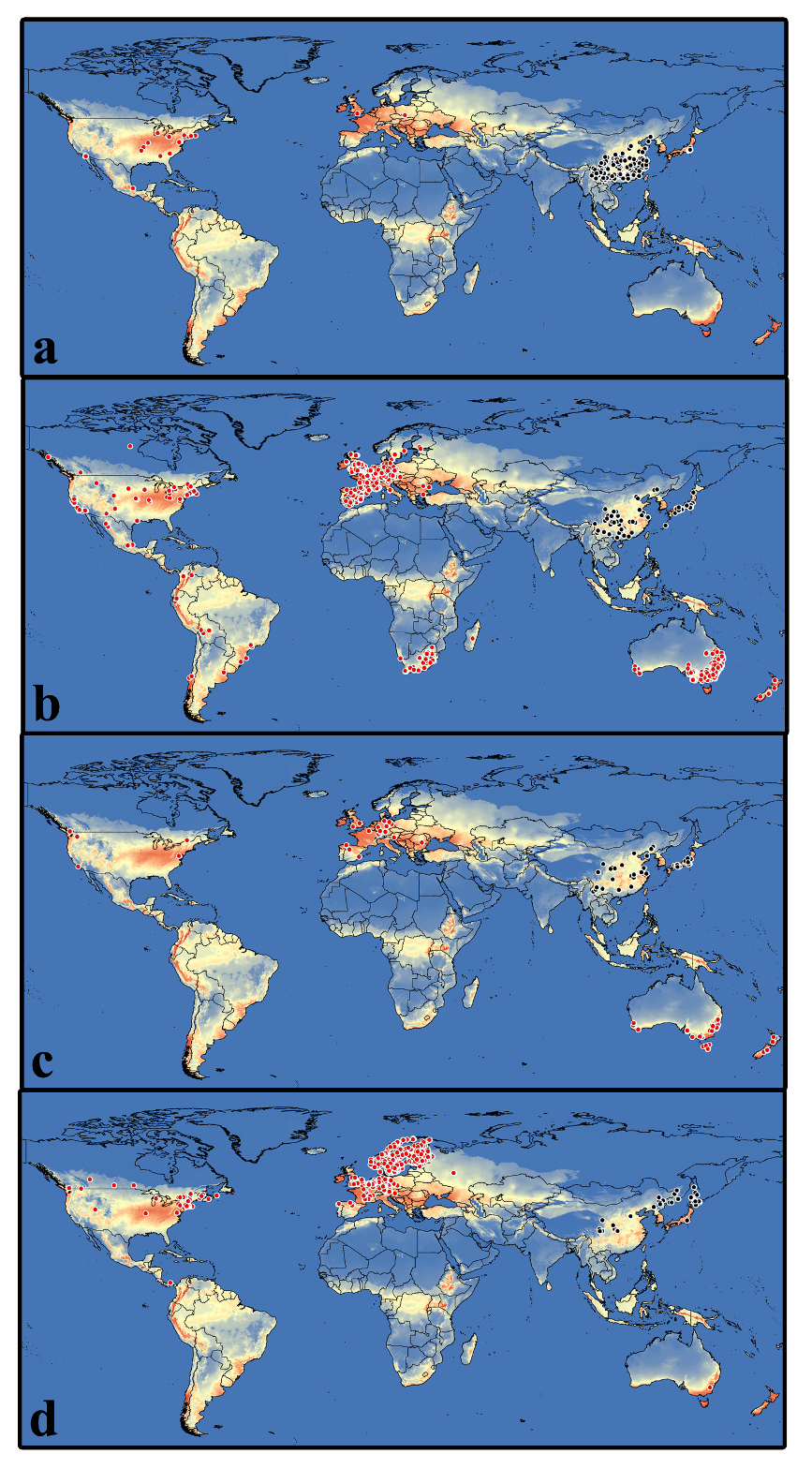

Supplement: Additional file 12: Figure S8. — Coordinates of four host plants’ positions on the map of predicted invasive areas of A. obscuriceps. Dark red color represents higher suitability, while dark blue indicates lower suitability. Black and red dots represent the native and invasive records respectively. (a) Castanea mollissima; (b) Salix babylonica; (c) Salix matsudana; (d) Sorbaria sorbifolia. (DOC 1211 kb) [file 12862_2016_748_MOESM12_ESM.doc]
